# Mangiferin: a natural miracle bioactive compound against lifestyle related disorders

**DOI:** 10.1186/s12944-017-0449-y

**Published:** 2017-05-02

**Authors:** Muhammad Imran, Muhammad Sajid Arshad, Masood Sadiq Butt, Joong-Ho Kwon, Muhammad Umair Arshad, Muhammad Tauseef Sultan

**Affiliations:** 1 Department of Diet and Nutritional Sciences, Imperial College of Business Studies, Lahore, Pakistan; 2 National institute of Food Science and Technology, University of Agriculture Faisalabad, Faisalabad, Pakistan; 3 Institute of Home and Food Sciences, Government College University, Faisalabad, 36000 Pakistan; 4 School of Food Science and Biotechnology, Kyungpook National University, Daegu, 41566 Republic of South Korea; 5 Department of Food Science and Technology, Bahauddin Zakariya University, Multan, Pakistan

**Keywords:** Bioactive molecules, Human cancers, Mangiferin, Nutrition, Health claims, Toxicity

## Abstract

The current review article is an attempt to explain the therapeutic potential of mangiferin, a bioactive compound of the mango, against lifestyle-related disorders. Mangiferin (2-β-D-glucopyranosyl-1,3,6,7-tetrahydroxy-9H-xanthen-9-one) can be isolated from higher plants as well as the mango fruit and their byproducts (i.e. peel, seed, and kernel). It possesses several health endorsing properties such as antioxidant, antimicrobial, antidiabetic, antiallergic, anticancer, hypocholesterolemic, and immunomodulatory. It suppresses the activation of peroxisome proliferator activated receptor isoforms by changing the transcription process. Mangiferin protects against different human cancers, including lung, colon, breast, and neuronal cancers, through the suppression of tumor necrosis factor α expression, inducible nitric oxide synthase potential, and proliferation and induction of apoptosis. It also protects against neural and breast cancers by suppressing the expression of matrix metalloproteinase (MMP)-9 and MMP-7 and inhibiting enzymatic activity, metastatic potential, and activation of the β-catenin pathway. It has the capacity to block lipid peroxidation, in order to provide a shielding effect against physiological threats. Additionally, mangiferin enhances the capacity of the monocyte-macrophage system and possesses antibacterial activity against gram-positive and gram-negative bacteria. This review summarizes the literature pertaining to mangiferin and its associated health claims.

## Background

**Fig. 1:**
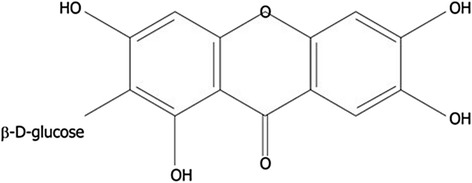
Chemical structure of mangiferin

Mangiferin is a xanthone present in significant levels in higher plants and in different parts of the mango fruit, such as the peel, stalks, leaves, barks, kernel, and stone. It is a promising antioxidant with tremendous health-related properties such as antiviral, anticancer, antidiabetic, antioxidative, antiaging, immunomodulatory, hepatoprotective and analgesic effects [1]. Isomangiferin and homomangiferin, which constitute 10% of the total phenolics, are also present in different parts of the mango tree, such as leaves, mango peel, and twigs, [2, 3]. They prevent the production of hydroxyl radicals due to their iron chelating ability in Fenton-type reactions [4]. The chemical structure of mangiferin is shown in Fig. 1.

Methanolic mangiferin extracts are potent antioxidants (EC_50_: 5.8 ± 0.96 μg/ml), and have demonstrated hepatoprotective actions against carbon tetrachloride-induced liver damage, further supporting the performance of their in vivo radical scavenging system [1]. Mangiferin also lowered hydrogen peroxide-induced lipid peroxidation in human peripheral blood lymphocytes in a dose-dependent manner [5]. A study conducted by [6] determined that the administration of mangiferin (1, 10, 100 μg/ml) significantly increased the resistance of erythrocyte cells to hydrogen peroxide-induced reactive oxygen species (ROS). It also prevented the depletion of GTP and total nucleotides (NT), as well as ATP damage, and restored energy charge potential in H_2_O_2_-treated erythrocytes in a dose-dependent manner. Mangiferin protects hepatocytes from free radical-mediated hypoxia/reoxygenation injury by forming mangiferin: Fe^3+^ complexes and neutralizing free radicals [7]. This iron-complexing ability of mangiferin has been shown to provide protection from lipid peroxidation induced by Fe^2+^-citrate in rat liver mitochondria. Ten micromolar mangiferin was able to protect against the loss of mitochondrial trans-membrane potential (ΔΨ) and the swelling of mitochondria in livers treated with 50 μM Fe^2+^-citrate. It also suppressed iron citrate-induced mitochondrial antimycin A-insensitive oxygen consumption, stimulated oxygen consumption, and prevented Fe^3+^ reduction of ascorbate due to Fe^2+^ auto-oxidation. Mangiferin is unable to participate in Fenton-type reactions and lipid peroxidation, as the absorption spectra of mangiferin: Fe^2+^/Fe^3+^ complexes indicates that the more stable Fe^3+^ complex is formed preferentially, and this compound cannot participate in the aforementioned mechanisms [8].

The Friedel-Crafts reaction promotes mangiferin synthesis, wherein the electron-rich aromatic compound and glycosyl donor are connected via glycosidic linkages [9]. Similarly, without the function of the C-9 carbonyl, aryl C-glycosylation is required due to electron deficiency [10, 11]. Mangiferin is synthesized through the hydrolysis of aglycone 1,3,6,7-tetrahydroxy-xanthone with R-acetobromoglucose, followed by a reaction entailing the formation of O-glycosidic bonds [12].

Nevertheless, the C-glucosyl bonds of several C-glucosides interact with the intestinal microflora, facilitating molecule metabolism and transformation into corresponding aglycones. Mangiferin can be used at low concentrations prevent cell damage through reducing localized oxygen concentrations, removing hydroxyl radicals and oxo-ferryl groups, producing mangiferin metal complexes, and initiating the reaction by which free radicals and polymer chain modulation are neutralized [13].

The chemical configuration of mangiferin is influenced by the antioxidant activity of phenolic compounds. Mangiferin showed higher iron chelating ability because of its great antioxidant potential, and its antioxidant potential mainly depends upon the type of compounds, purity of active compounds, applied methods, and higher degree of polymerization [10]. Flavonoids have lower IC_50_ values because of a 2-3 double bond conjugated with a 4-carbonyl group present in its microsomal system, which improved the phenoxyl radical-stabilizing effect of 3,4-catechol [14, 15]. Moreover, flavonol glycosides exhibit antioxidant activities due to the presence of glycosidic moieties [16]. Other chemical modifications like O-glycosylation and methylationin the flavonoids thereby changing the entire structure of the bio-molecules [17]. The activity levels of tricarboxylic acid (TCA), as well as key enzymes in the electron transport chain, such as malate dehydrogenase, isocitrate dehydrogenase, succinate dehydrogenase, and α-ketoglutarate dehydrogenase, are decreased by mangiferin in rats with lung cancer [18]. Mangiferin was also shown to be effective in vitro against glycated protein/chelate iron-induced toxicity in human umbilical vein endothelial cells, as it decreased lipid peroxidase and increased antioxidant enzymes levels [19].

Mangiferin has also been shown to exert a pro-hypoglycemic role by modulating glucose metabolism, ameliorating insulin resistance, lowering cholesterol synthesis, and inhibiting the expression of the tumor necrosis factor α and inducible nitric oxide synthase [20]. It also induces apoptosis and inhibits the progression and promotion of cell proliferation by interfering with cell cycle regulation, the signaling of several cancer transduction pathways in tumor cells [21].

Jutiviboonsuk and Sardsaengjun [22] estimated the mangiferin content in the methanolic extract of the Nam Doc Mai variety to be 2.80 g/100 g, followed by the Keow Savoey variety at 2.40 g/100 g, and Gaew variety at 1.30 g/100 g.

## Mangiferin synthesis and metabolism

Synthesis of mangiferin is accomplished by the friedel-Craft reactions, wherein the glycosyl donor and the electron rich aromatic compound are linked by glycosidic linkage [23]. In this case, aryl C-glycosylation of non-functional C-9 carbonyl groups is necessary because of the lack of electrons [10, 11]. In the presence of sodium methoxide (NaOMe), mangiferin gives 0.1% yield followed by the formation of an O-glycosidic bond through hydrolysis by treatment of the aglycone 1,3,6,7-tetrahydroxyxanthone with R-acetobromoglucose [12, 24]. Mangiferin is synthesized by the reaction of aglycone 1,3,6,7-tetrahydroxyxanthone with R-acetobromoglucose followed by hydrolysis of the formed O-glycosidic linkage [12]. Aglycone compounds have higher antioxidant potential compared to their corresponding glycosides [25].

In C-glucosides, the anomeric oxygen atoms present in glucose are substituted with carbon atoms, making this family significantly dissimilar from the O-glycosides [26]. They are stable against enzymatic and acid degradation [27]. In humans, the intestinal flora plays many important roles in the metabolism of various substances. Many C-glucosides contain C-glucosyl bonds, including abrusin 2-O-β-D-apioside, mangiferin, aloesin, aloeresin A, bergenin, barbaloin, puerarin, safflor yellow B, and homoorientin, and are also transformed by human intestinal bacteria into the corresponding aglycones [28]. Importantly, the glycosyl substituent has antioxidant advantages, owing to its structure and position, over the aglycone substituent [17]. The bioavailability of glycosides is enhanced through the glucose moiety [29].

## Pharmacokinetic role

Mangiferin is obtained from the mango with many other active ingredients but poor lipophilicity and hydrophilicity. Mangiferin concentrations were measured using RP-HPLC in rats orally dosed with crude mangiferin and a mangiferin-phospholipid complex. The results showed that mangiferin-phospholipid complex solubility in water and n-octanol was improved, and the complex oil-water partition coefficient was improved by 6.2 times. Furthermore, significantly enhanced intestinal permeability was observed in the rats. AUC and peak plasma concentration in rats treated with crude mangiferin were lower than those treated with the mangiferin-phospholipid complex [30].

Hepatoprotective activity was increased by mangiferin complex administration in rats treated with carbon tetrachloride through restoration of liver antioxidant and serum hepatic marker enzymes. The bioavailability of mangiferin in rats increased 9.75-fold when using the mangiferin complex compared to pure mangiferin. Moreover, hepatoprotection in rats was increased via administration of a mangiferin-soya phospholipid complex due to improved bioavailability [31]. Zhang et al [32] determined that mangiferin recovery ranged from 80.0 to 85.6% in the eye and from 82.0 to 88.0% in the plasma. Mangiferin can pass through the blood-ocular barrier and has been shown to be effective in curtailing various eye diseases [32]. Similarly, [33] investigated the concentration of mangiferin in plasma (0.6 to 24 μg/mL) and urine (0.48 to 24 μg/mL) through HPLC.

Likewise, mangiferin concentration in feces and urine were 1.4 and 1.6% for 9 and 11 days respectively after the administration of 74 mg mangiferin/kg body weight [34]. Using high-speed countercurrent chromatography via a HPLC-diode array detector, mangiferin and neomangiferin were quantified as 292.8 mg and 165.6 mg from an n-butanol:water (1:1, v/v) extract of the Chinese medicinal plant *Anemarrhena asphodeloides* Bunge [35].

Furthermore, researchers have quantified the mangiferin, neomangiferin and 5-hydroxymethyl furancarboxaldehyde (5-HMF) content from the Chinese medicinal plant *Anemarrhena asphodeloides*. They quantified 166.0 mg and 293.0 mg of the neomangiferin and mangiferin respectively with 99.0 and 99.6% purity through on-line high-speed countercurrent chromatography and HPLC-diode array detection coupling [35]. In another study, [36] determined that the mangiferin concentration in the retina 0.45 h after intravenous administration of a 50 mg/kg dose was 6.00 ± 1.50 μg/ml, which gradually decreased to 0.30 ± 0.02 μg/ml at 5 h.

## Health perspectives

### Anticancer

Taking precautions against carcinogens has become a very effective anticancer preventive measure. Fruits and vegetables have been shown to have anticancer potential owing to the presence of bioactive compounds. Mangiferin shows antileukemic and preventive effects in HL-60 leukemia cells. After treatment, mangiferin caused cell cycle arrest in the G2/M phase. It accomplished this in a dose-dependent manner at higher concentrations, and further induced *Wee1* mRNA expression while significantly suppressing mRNA expression of *Chk1* and *cdc25C*. It also significantly inhibited ATR, Chk1, Wee1, Akt, and Erk1/2 phosphorylation. In addition, mangiferin-treatment also decreased activation of cyclin B1 and cdc25C, as well as protein expression levels of Akt and Wee1. It also inhibited cell cycle progression via ATR-Chk1 DNA damage stress-response pathways, leading to cell cycle-arrest in leukemia cells at the G2/M phase [37]. Mangiferin targets several proinflammatory transcription factors, growth factors, cell-cycle proteins, cytokines, kinases, adhesion molecules, chemokines, and inflammatory enzymes by inhibiting the initiation, promotion, and metastasis stages of cancer [38]. Estrogen receptor alpha (ERα) and beta (ERβ) are two regulators of gene transcription responsible for breast cancer. Mangiferin activates the estrogen receptor alpha (ERα) [39].

In U87 glioma cells, miR-15b regulates the expression of matrix metalloproteinase (MMP)-9. Mangiferin significantly restrained proliferation and increased apoptosis through promotion of miR-15b and inhibition of MMP-9. Addition of an MMP-9 agonist antibody in U87 glioma cells reduces the efficacy of mangiferin [40]. Dilshara et al. [41] determined that mangiferin substantially lowered TNFα-induced MMP-9 activity, decreased nuclear factor-κB (NF-κB) activity, and suppressed nuclear translocation of the NF-κB subunits p65 and p50 in androgen-sensitive human prostate adenocarcinoma cells.

Lung carcinoma was induced by benzo(a)pyrene (BaP) (50 mg/kg body weight (BW)) in healthy male Swiss albino mice, resulting in a decrease in body weight, lung weight, and enhanced liver marker enzymes. However, administration of mangiferin (100 mg/kg BW) dissolved in corn oil in these animals lowered the activity of lysosomal enzymes such as β-glucuronidase, acidphosphatase, β-galactosidase and N-acetyl glucosaminidase [42]. Mangiferin significantly lowered DNA damage in etoposide-treated mononuclear cells (MNCs), promoted Nrf2 translocation into the nucleus, enhanced nuclear Nrf2 expression, upregulated NAD(P)H dehydrogenase [quinone] 1 (NQO1), an Nrf2 signaling target, and increased Nrf2 binding with NQO1-antioxidant response element (ARE) sequences in human umbilical cord mononuclear blood cells [43]. In the human cell line nasopharyngeal carcinoma (CNE2 cells), mangiferin different concentrations are (12.5, 25, 50, 100, 150 and 200 μM) suppressed CNE2 cell proliferation via G2/M phase cell cycle arrest, induced early apoptosis, downregulated the mRNA and protein levels of Bcl-2, and upregulated Bax [44]. It also has been shown to inhibit NF-κB (p65 subunit) and IκBα expression in peripheral blood mononuclear cells (PBMCs) in rats with cigarette smoke-induced chronic bronchitis. It considerably lowers the serum high-sensitivity C-reactive proteins (Hs-CRP) and TNFα levels, and reduced chronic inflammatory damage to the bronchioles in PBMCs [45].

The higher concentrations of mangiferin (10, 25 or 50 μM) with doxorubicin for 96 h have the ability to re-sensitize MCF-7 breast cancer cellsthrough reducing cell viability and inhibiting P-glycoprotein (P-gp) expression [46]. Mangiferin increases Nrf2 expression and protein stability in human HL60 myeloid leukemia cells in vitro in a dose- and time-dependent manner. Moreover, it also inhibits the ubiquitination and degradation of blood cells through increased stability of the Nrf2 protein [47].

In HL-60 cells, mangiferin (50 μM) enhanced Nrf2 protein accumulation, increased Nrf2 binding of ARE, regulated NQO1 expression, and decreased intracellular ROS levels. It also relieved oxidative stress and attenuated etoposide-induced cytotoxicity in mononuclear human umbilical cord blood cells [48].

Finally, mangiferin has been shown to lower cell viability, suppress metastatic potential, decrease MMP-7 and -9 expression, reverse epithelial-mesenchymal transition (EMT), and inhibit the β-catenin pathway in breast cancer cell lines. Mangiferin considerably decreased tumor proliferation, weight, and volume, and enhanced apoptosis, as well as also decreased the expression levels of MMP-7, MMP-9, active β-catenin, vimentin, while increasing the expression of E-cadherin in in vitro MDA-MB-231 xenograft mice [49].

In another study, [50] determined that different concentrations of mangiferin (25–200 μM) exhibited antiproliferation effects on K562 cells in a dose-dependent manner through downregulating expression of the *BCR* and *ABL* genes. Likewise, mangiferin (80 μM) also showed inhibitory effects on HL-60 cells by stopping the progression of cell cycle at the phase of G2/M, and enhancing the expression of *CDC2* and *CCNB1* mRNA [51]. Mangiferin has an antiproliferative role on the telomerase activity of K562 cells by inducing apoptosis and upregulating Fas levels [52].

**Fig. 2:**
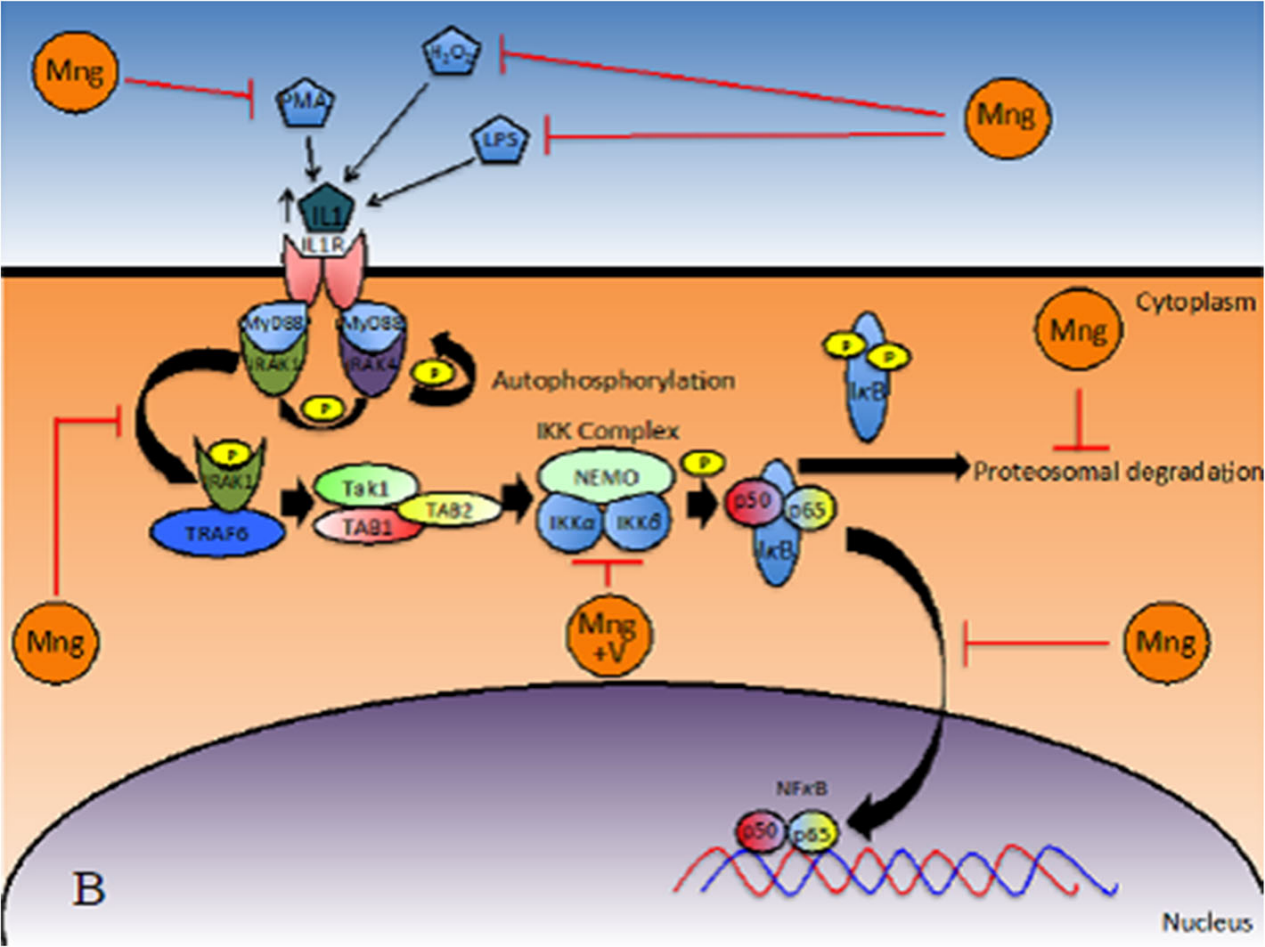
Inhibition of NFκB via the (A) classical and (B) alternative pathways by mangiferin (adapted from [11, 45, 55, 123, 124])

Oral administration of mangiferin (100 miligram/kilogram BW) through the diet for 18 weeks significantly ameliorated the elevated levels of glycoprotein components, membrane lipid peroxidation, and ATPases in lung carcinoma-induced animals. Mangiferin increased the concentration of glutathione, catalase (CAT), superoxide dismutase, glutathione reductase, glutathione peroxidase, vitamin E, and vitamin C [53]. Similarly, in the breast cancer cell line MDA-MB231, mangiferin also suppressed classical NFκB activation by IκB kinases (IKK) α/β in impairing IκB degradation, NFκB translocation, and NFκB/DNA binding. Furthermore, mangiferin inhibits additional NFκB pathways which participated in cancer cell survival and therapy resistance such as c-Jun N-terminal kinases (JNK) 1/2, MEK1, p90 ribosomal s6 kinase, and mitogen- and stress-activated protein kinase 1 [54]. Mangiferin has the potential for antigenic toxicity of neurotoxicity against methylmercury (MeHg) -induced IMR-32 (human neuroblastoma) cell lines. It considerably suppresses MeHg-induced DNA damage, lowers MeHg-induced oxidative stress, intracellular Ca^2+^ influx, and inhibits depolarization of the mitochondrial membrane. It also enhances glutathione (GSH) and glutathione S-transferases (GST) levels, leading to significantly decreased malondialdehyde (MDA) formation [55]. It also induced apoptosis in the human acute myeloid leukemia cell line HL-60 through comcomitant enhancement of caspase-3 activity and DNA fragmentation, significantly decreasing the nuclear entry of NF-κB p65, suppressing NF-κB activation, and inhibiting the expression of Bcl-xL and X-linked inhibitor of apoptosis protein (XIAP) [56]. The inhibition of NFκB via the (A) classical and (B) alternative pathways by mangiferin is shown in Fig. 2.

## Antinflammatory activity

Acute nociceptive, neuropathic pain and inflammatory responses depend on the peripheral activation of primary sensory afferent neurons, which show several inhibitory neuro-receptors such as opioid, alpha-adrenergic, cholinergic, cannabinoid receptors, and adenosine and these receptor agonists represent possible targets for drugs [57, 58].

Mangiferin protects against peptidoglycan- or lipopolysaccharide (LPS)-stimulated peritoneal macrophages via inhibition of interleukin (IL)-1 receptor-associated kinase 1 (IRAK1), NF-κB and mitogen-activated protein kinase (MAPK) phosphorylationAdministration of mangiferin (10 μM) inhibited LPS-stimulated expression of TNF-α, IL-6, and IL-1β in macrophages by 81.0, 88.3 and 90.0% respectively, and it also enhanced the expression of IL-10 by 132.0% compared to untreated controls. It also suppressed colon shortening, 2,4,6-trinitrobenzenesulfonic acid (TNBS)-induced IRAK1 phosphorylation, NF-κB activation, colonic myeloperoxidase activity, TNBS-induced cyclooxygenase-2 (COX-2) upregulation, and induction of nitric oxide synthase in a murine TNBS-induced colitis model. Similarly, mangiferin (20 mg/kg) considerably suppressed TNF-α (78%), IL-1β (82%), and IL-6 expression (88%) but induced the IL-10 expressions to 79% of the normal control group. It also prevented inflammatory disorders (i.e. colitis) by regulating MAPK and NF-κB/IRAK1 through the inhibition of phosphorylation of signaling pathways [59].

Mangiferin has an anti-asthmatic role against Th1/Th2 cytokines through multiple mechanisms such as: (1) reducing eosinophil and total inflammatory cell infiltration, (2) decreasing prostaglandin (PG) D2 in bronchoalveolar lavage fluid (BALF) and ovalbumin-specific immunoglobulin (Ig) E in serum, (3) downregulating the levels of cytokines/chemokines such as Th2-related IL-3, IL-4, IL-5, IL-9, IL-13, IL-17, TNF-α, and RANTES, and (4) increased serum Th1-related cytokine expression such as interferon (IFN)-γ, IL-2, IL-10 and IL-12. Additionally, it has also been demonstrated to reduce abnormal mRNA levels of both Th1 (IL-12 and IFN-γ) and Th2-type cytokines (IL-4, IL-5 and IL-13), as well as attenuate the Th1/Th2 cell ratio imbalance, and suppress the activation and expression of Trans-acting T-cell-specific transcription factor GATA-3 and (signal transducer and activator of transcription (STAT-6) in excised lung tissues. The oral administration of mangiferin (12.5-50 mg/kg) for 7 days lowered flinching, licking/biting behaviors, and decreased formalin-induced long-term damage secondary to chronic mechano-hyperalgesia [60]. Mangiferin suppressed LPS-induced IL-6 production and cystathionine-b-synthase (CBS) expression after 6 and 24 h in the hippocampus [61]. Oral administration of mangiferin (10 to 100 mg/kg) significantly exhibited antinociceptive activity against chemogenic pain in formalin- and capsaicin-induced neuro-inflammatory pain, and acetic acid-induced visceral pain in mouse model of experimental pain [62].

Mangiferin also attenuates mortality and acute lung injury (ALI) induced by cecal ligation and puncture. It also inhibited NF-κB signaling and sepsis activated-MAPKs, suppressing the production of proinflammatory mediators. It also upregulated the expression and activity of heme oxygenase (HO)-1 in the lung of septic mice in a dose-dependent fashion [63]. Bhatia et al. [64] determined that mangiferin (1–50 μM) reduced 8-isoPGF2α and PGE2 production induced by LPS in a dose-dependent manner in rat primary microglia. It also lowered COX-2 protein synthesis induced by LPS without modifying the transcription of COX-2.

Mangiferin also ameliorates intestinal inflammation and impairs the gastrointestinal transit postoperative ileus (POI) of rats. It recovers delayed intestinal transit induced by intestinal manipulation. Furthermore, mangiferin considerably suppressed myeloperoxidase activity (a marker of neutrophil infiltration) and nitrate/nitrite ratio, as well as lowered the plasma levels of IL-1β, TNF-α, IL-6, and monocyte chemoattractant protein-1 (MCP-1) in rats [65].

Mangiferin also downregulates the mRNA expression of pro-inflammatory mediators such as induced nitric oxide synthase (iNOS), TNF-α, IL-1β, and IL-6, phosphorylation of NF-κB p65, and intercellular adhesion molecule-1 (ICAM-1), in the colon of a dextran sulfate sodium (DSS)-induced colitis model in mice. It also inhibited DSS-induced MAPK protein phosphorylation/activation and also blocked TNF-α stimulated RAW 264.7 mouse macrophage cell nuclear translocation of NF-κB. It also suppressed NF-κB transcriptional activity in HT-29 human colorectal adenocarcinoma cells in a dose-dependent manner [66]. Furthermore, mangiferin shows a gastroprotective effect in mice. Administration of mangiferin (3, 10 and 30 mg/kg) decreased ethanol-induced gastric damage by 30, 35, & 63% respectively, and reduced indomethacin-induced gastric damage by 22, 23, and 57% respectively. In pylorus-ligated rats, gastric secretion and total acidity significantly decreased by mangiferin, and effectively prevents the depletion of the ethanol-related protein from the gastric mucosa of the non-protein sulfhydryl content [67].

## Diabetes treatment

Diabetes is becoming a major health disorder among the population, and more than 80% of diabetic patients suffer from type 2 diabetes. This type of diabetes is associated with a reduced ability to increase the glucose utilization in its major target tissues: skeletal muscle and adipose tissue. These increases in blood glucose and insulin levels are linked with cardiovascular diseases (hypertension, retinal injury, and atherosclerosis), fatty liver, dyslipidemia, and renal diseases [68].

Eight weeks of mangiferin treatment significantly lowered plasma glucose and triglyceride (TG) levels in db/db mice. It enhanced pancreatic β cell mass and amount of glucose and insulin uptake along with increased the phosphorylation of AMP-activated protein kinase (AMPK) in n 3 T3-L1 cells. It also activated the AMP-activated protein kinase (AMPK) phosphorylation of AMPK and activated along its downstream target, acetyl -CoA carboxylase (ACC) in the liver, hypothalamus, muscle and adipose tissue of C57BL/6 mice [69]. Likewise, the oral administration of mangiferin (20 mg/kg, intraperitoneal administration (i.p.)) for 4 weeks in streptozotocin-induced hyperglycemic rats improved insulin sensitivity, modulated lipid profile, and reverted adipokine levels [70]. Similarly, different concentrations of mangiferin (15, 30, and 60 mg/kg/day oral administration) were given to diabetic rats for 9 weeks, resulting in a reduction of osteopontin production, kidney inflammation, and renal fibrosis. Chronic treatment with mangiferin prevented renal glomeruli fibrosis, as well as decreased α-smooth muscle actin and collagen IV expression in the diabetic rat. In contrast, this also reduced the expression of osteopontin in the renal-cortex of diabetic rats, COX-2 and NF-κB P65 subunits. Finally, mangiferin reduced interleukin-1β in the serum and kidneys of diabetic rats [71].

Magniferin has also been shown to lower ROS production and decrease intracellular antioxidant defenses, and mediates diabetic nephropathy, as it modulates the MAPK (P38, JNK and ERK1/2), PKC isoforms (PKCα, PKCβ and PKCε), TGF-β1 pathways, as well as the NF-κB signaling cascades involved in this pathophysiology [72]. Wang et al. [73] determined that mangiferin exhibited an antidiabetic role in adult C57BL/6 J mice. The administration of mangiferin (30 and 90 mg/kg BW) improved glucose tolerance and glycemia, increased β-cell hyperplasia and serum insulin levels, lowered β-cell apoptosis, elevated β-cell proliferation and upregulated cyclin D1, D2 and cyclin-dependent kinase 4 (Cdk4) at 7–14 days post-partial pancreatectomy. Additionally, mangiferin promoted β cell regeneration and the expression of pancreatic and duodenal homeobox gene 1 (PDX-1), glucose transporter 2 (GLUT-2), neurogenin 3 (Ngn3), glucokinase (GCK), and Forkhead box protein O1 (Foxo-1).

Mangiferin (15, 30 and 60 mg/kg) treatment for nine weeks significantly improved chronic renal insufficiency of diabetic rats, and was demonstrated to reduce kidney weight index, albuminuria, glomerular extracellular matrix expansion, blood urea nitrogen, and glomerular and accumulation basement membrane thickness. Meanwhile, it also enhanced the enzymatic activity, as well as the protein and mRNA expression of Glo-1, as well as decreased the mRNA and protein expression of advanced glycation endproducts (AGEs) and receptor for advanced glycation end products (RAGE) receptor in diabetic rat renal cortex. Moreover, it also reduced the concentrations of malondialdehyde (MDA) and enhanced the concentrations of glutathione in the diabetic rat kidney [74].

Mangiferin (10 and 20 mg/kg) administered once daily for 28 days in STZ-induced diabetic rats exhibited anti-diabetic activity. It significantly reduced plasma low-density lipoprotein cholesterol LDL-C) and TG levels, lowered total cholesterol level and increased high-density lipoprotein cholesterol (HDL-C) levels. Furthermore, the atherogenic index of diabetic rats decreased and mangiferin improved oral-glucose tolerance in normal rats loaded with glucose [75].

In diabetic nephropathy rats, mangiferin considerably lowered the serum levels of advanced glycation end products, red blood cell sorbitol concentrations, malonaldehyde level, 24 h albuminuria excretion, and enhanced serum antioxidant enzymes such as superoxide dismutase and glutathione peroxidase. In addition, it inhibits the expansion of glomerular extracellular-matrix and diabetic nephropathy rat glomeruli TGF-β1 accumulation and transformation, high glucose-induced mesangial cell proliferation and mesangial cells collagen IV in a glomerular diabetic nephropathy rat model [76]. The health perspectives of mangiferin is shown in Table 1.

**Table 1:** Health perspectives of mangiferin

Disorders	Mechanisms	Reference
Anticancer	Inhibition of *cdc25C* and *Chk1* mRNA expression Inhibition of Erk1/2, Chk1, ATR, Akt, and Wee1, phosphorylation cdc25C and cyclin B1 activation decreased Progression of cell cycle inhibited through ATR-Chk1 DNA damage stress-response pathways	[37]
	Apoptosis increased and proliferation restrained	[40]
	Activity of TNFα-induced MMP-9 decreased, lowering the activity of NF-κB (nuclear factor-κB) Suppressed p50 and p65	[41]
	Lowering β-glucuronidase, acidphosphatase, β-galactosidase and N-acetyl glucosaminidase activity	[42]
	Proliferation of CNE2 cells was inhibited by cell cycle arrest at G2/M phase Down-regulates levels of mRNA and Bcl-2 protein, and up-regulates Bax	[44]
	Serum-reduced TNFα and Hs-CRP (high-sensitivity C-reactive proteins) levels	[45]
	Inhibiting the expression of P-glycoprotein (P-gp)	[46]
	Increased stability of protein and expression of Nrf2	[47]
	Increased accumulation of Nrf2 protein, Nrf2 binding enhanced in ARE, regulated expression of NQO1	[48]
	Reduced expression of MMP-7 and -9, reversed EMT (epithelial-mesenchymal transition) Inhibition of the beta-catenin pathway	[49]
	Enhanced *CDC2* and *CCNB1* mRNA expression	[51]
	Up regulating the Nrf2 signaling target, and NAD(P)H dehydrogenase [quinone] 1 (NQO1)	[43]
	Up regulated levels of Fas	[52]
	Estrogen receptor alpha (ERα) activated	[39]
	Classical NFκB activation suppressed by IκB kinases (IKK) α/β Additional NFκB-pathways inhibit	[54]
	Inhibition of MeHg-induced DNA damage Lowering the intracellular Ca^2+^ influx, MeHg-induced oxidative stress Mitochondrial membrane depolarization inhibits	[55]
	Significantly reduced NF-κBp65 nuclear entry, inhibition of NF-κB activation Inhibition of XIAP (X-linked inhibitor of apoptosis protein) and Bcl-xL expression	[56]
Anti-inflammatory activity	The expression of TNF-α, IL-6 and IL-1β was inhibited by LPS Increased expression of IL-10 Inhibition of colonic shortening, colonic myeloperoxidase activity, NF-κB activation, 2,4,6-trinitrobenzenesulfonic acid (TNBS)-induced IRAK1 phosphorylation Inhibition of IL-6, IL-1β and TNF-α (78%) expression	[59]
	Reduced total inflammatory cell infiltration and eosinophil Lowering the prostaglandin (PG) D2 Down regulated the levels of IL-3, IL-4, IL-5, IL-9, IL-13, IL-17, TNF-α and RANTES-related Th2 Th1-related cytokines expression increased in serum, such as IL-2, IL-10 and IL-12, interferon (IFN) -γ Reduced abnormal mRNA levels of both Th2-type cytokines (IL-4, IL-5 and IL-13) and Th1 (IL-12 and IFN-γ)	[60]
	Suppression of IL-6 production induced by LPS and expression of cystathionine-b-synthase (CBS)	[61]
	Inhibition of sepsis activated-MAPKs and NF-κB signaling Upregulated activity and hemo oxygenase expression (HO) -1	[63]
	Reduced PGE2 and 8-isoPGF2-alpha production Lowering the protein synthesis of COX-2	[64]
	Suppressed nitrite/nitrate ratio and myeloperoxidase activity Lowering the level of plasma of TNF-α, IL-1β, monocyte chemoattractant protein-1 (MCP-1) and IL-6	[65]
	Intercellular adhesion molecule-1 (ICAM-1) and NF-κB p65 phosphorylation down-regulated Transcriptional activity of NF-κB suppressed	[66]
	Significantly reduced total acidity and gastric secretion volume	[67]
Diabetes prevention	Glucose amount and pancreatic beta cell mass enhanced AMPK and the activation of AMP-activated protein kinase (AMPK) phosphorylation	[69]
	Sensitivity of insulin improved, lipid profile modulated and adipokine levels reverted	[70]
	TGF-β1 pathways, PKC isoforms (PKCα, PKCβ and PKCε), MAPK (P38, JNK and ERK1/2) modulated	[72]
	Lowering the β-cell apoptosis Upregulate the cyclin D1, cyclin D2 and Cdk4 (cyclin-dependent kinase 4) It promoted the regeneration of β-cells and the expression of duodenal and pancreatic homeobox gene 1 (PDX-1), glucose transporter 2 (GLUT-2), neurogenin 3 (Ngn3), glucokinase (GCK) and Forkhead box protein O1 (Foxo-1).	[73]
	Glo-1 mRNA expression, protein and enzymatic activity enhanced Reduced mRNA and protein expression of receptors in advanced end products of glycation (AGEs) and advanced end products of glycosylation (RAGE) receptors	[74]
	Reduce the concentration of RBCs sorbitol, malonaldehyde level Inhibition of expansion of the glomerular extracellular matrix, and accumulation and transformation of growth factor-beta 1 over-expression	[76]
	Preventing renal glomeruli fibrosis Reduced the expression of alpha-smooth muscle collagen IV and actin Reduced COX-2, NF-κB p65 and osteopontin subunit expression Lowering the interleukin-1β	[71]
Cardiovascular preventive role	The attenuated expression of NLRP3 and TXNIP, reduced production of IL-1β and IL-6, and the inhibited inhibition of the inflammatory activation of TXNIP/NLRP3 Mitochondrial Δψ restored Activity of caspase-3 suppressed Improved inhibition of ET-I secretion and NO production reduced Increased AMPK inhibitor compound C and phosphorylation of AMPK Inhibition of inflammation induced by TXNIP/NLRP3 activation related to stress in the endoplasmic reticulum of the endothelial cell	[78]
	Reduction of serum lactate dehydrogenase (LDH) and creatine kinase levels, reduction of MDA levels	[79]
	Reduce diabetic cardiomyopathy (DCM), and to prevent the accumulation of collagen in the heart	[80]
	Improved effect of pathological changes induced by pH Reduction of the formation of lipid peroxides and retention of cardiac markers activity	[81]
	Mangiferin plays an important role in the reduction of triglyceride, free fatty acid (FFA) and cholesterol levels in both heart and serum and may also increase phospholipid levels of cardiac tissue in isoproterenol-induced cardiotoxic rats	[82]
	Lysosomal integrity preserved	[81]
Oxidative stress	Blood sugar reduced, elevated levels of plasma insulin and antioxidant enzymes increased such as CAT, glutathione peroxidase (GPx) and SOD	[85]
	Apoptotic cells induced and the normalized potential of the mitochondrial membrane and cellular-antioxidant levels	[94]
	Prevention of Ca2 + -induced depletion of antioxidant enzymes	[95]
	Cadmium-induced secretion of both IL-8 and IL-6 prevented	[38]
	Regulated production of Nrf2 and NLRP3	[87]
	MnNCE and MnPCE ratio reduced, and increased the ratio of PCE/NCE	[92]
	MGLUT9 (murine glucose transporter 9) and uric acid transporter 1 (mURAT1) mRNA and protein levels were down-regulated Upegulated the murine organic anion transporter 1 (mOAT1) Increased renal organic cation levels, as well as carnitine transporter (mOCTN1, mOCTN2, mOCT1 and mOCT2) expression levels	[91]
	Decreased MDA levels, and content of TNF-α and IL-8 in lung tissues whereas the RAW264.7 macrophages COX-2 mRNA expression	[90]
	Prevented 6-hydroxydopamine (6-OHDA)-induced cell death Decreased the levels of IL-6 and MDA	[89]
	Inhibition of Pb(II)-induced mitogen-activated protein kinases (MAPKs) activation (phosphor-JNK phospho- p38, phospho-ERK 1/2), NF-κB nuclear translocation and apoptotic cell death.	[122]
Neuro-protective role	Decreased inflammatory cytokines levels, oxidative stress marker levels and hippocampal brain derivd neutrophic-factor (BDNF) content.	[96]
	Prohibited dopamine depletion, and MPTP-induced interactive deficits	[97]
	Constrain tracheal reductions Increased protein levels of cGMP and nitric oxide synthase at the cellular level Eradicated the growth in cGMP levels	[98]
	Improved cellular responses, antigen-specific IgM levels, and lymphoid organ weights	[75]
	Induced a significant increase in supernatant levels of nerve growth factor and TNF-α	[100]
	Prohibited from improved IL-1β and glucocorticoid (GC) plasma levels, and loss of redox balance and reduction in catalase brain levels Prevented from increase in pro-inflammatory mediators such as TNF-α, NF-κB, TNF receptor 1, as well as synthesis enzymes such as iNOS and COX-2	[99]
Hyperlipidemia preventive strategy	Regulated the metabolic pathways such as the glyoxylate, tricarboxylic acid (TCA)and taurine cycles	[101]
	Lowered the levels of proteins which are critical for lipogenesis, such as acetyl-CoA carboxylase 1 (Acac1) gene and fatty acid stearoyl-CoA desaturase 1 (Scd1)	[102]
	Increased cell viability, improved mitochondrial membrane potential	[103]
	Increased glucose and pyruvate oxidation and ATP production	[104]
	Protected from the mitochondrial NAD(P)H-linked substrates depletion and NADPH spontaneous oxidation	[105]
	Upregulated mRNA expression of PPAR-α, carnitine palmitoyltransferase 1 (CPT-1), and fatty acids (CD36) Decreased the mRNA expression of sterol regulatory element binding protein 1C (SREBP-1c), diacylglycerol acyltransferase 2 (DGAT-2), acetyl coenzyme a carboxylase (ACC), and microsomal triglyceride transfer protein (MTP)	[15]
	Upregulate the bone morphogenetic protein (BMP)-2, BMP-4 and transforming growth factor (TGF)-β Upregulated containing gene 9 (SOX9), sex- influential region Y-box (SRY-box), aggrecan, type 2 α1 collagen (Col2α1) and cartilage link protein. Reversed the production of BMP-2, SOX9, BMP-4, TGF- β, Col2α, aggrecan and cartilage link protein Upregulated the phosphorylation of Smad 2, Smad 3, Smad 1/5/8, and SOX9 in IL-1β-stimulated MSCs	[107, 108]
Miscellaneous properties	Inhibited IgE production, anaphylaxis reaction, histamine-induced vascular permeability, histamine release, and the lymphocyte proliferative response	[109]
	Inhibit the expression of COX-2 and leukocyte adhesion and rolling	[118]
	Binding activity of DNA of AP-1 (activator protein-1) inhibit, a factor of transcription for MMP-1	[111]
	Protected normal human intestinal epithelial cells (HIECs) from radiation-induced injuries	[112]
	Inhibited nuclear factor (NF)-kappaB activation in scopolamine or TNF-alpha-stimulated BV-2 microglial cells	[113]
	Decreased expression of osteoclast gene markers such as calcitonin receptor, cathepsin K, V-ATPase d2 and DC-STAMP Suppressed RANKL-induced activation of NF-κB, as well as p65 nuclear translocation and IκB-α degradation	[114]
	Inhibited the passive cutaneous anaphylaxis (PCA) IgE-antigen complex Inhibited the expression of pro-inflammatory cytokines TNF-α, IgE-antigen complexes, of IgE switching cytokines, IL-4	[9]
	Possessed antibacterial activity against Bacillus pumilus and Salmonella agona	[116, 117]
	Ameliorated anxiety-like behaviour and also improved anhedonic behavior Attenuated neuroinflammation in the prefrontal cortex	[121]

## Cardiovascular protective role

Cardiovascular diseases such as congenital heart disease, cerebrovascular disease, thyroid abnormalities (hypo- and hyperthyroidism), hypertension, peripheral artery disease, diabetes mellitus (both types 1 and 2), heart failure, and rheumatic heart disease are the most prevalent diseases around the globe, and are responsible for high rates of morbidity and mortality [77].

In response to endoplasmic reticulum stress, thioredoxin interacting protein (TXNIP) expression increases, followed by activation of small NACHT, LRR and PYD domains-containing protein 3 (NLRP3) and increased secretion of IL-1β. Mangiferin reduced the production of free radicals and also inhibited ER stress-related oxidative stress by attenuating endoribonuclease inositol-requiring enzyme 1 (IRE1α) phosphorylation. It also attenuated NLRP3 and TXNIP expression, reduced IL-1β and IL-6 production, and aided inhibition of TXNIP/NLRP3 inflammatory activation. Mangiferin also prevented oxidative stress through multiple mechanisms, including (1) restoring mitochondrial Δψ, (2) suppressing caspase-3 activity, (3) protecting from apoptosis induced by high glucose, (4) ameliorating the inhibition of ET-1 secretion and reduction of NO production, and (5) increasing AMPK phosphorylation and AMPK inhibitor compound C diminished its beneficial effects. Mangiferin improved endothelial dysfunction by inhibiting endothelial cell endoplasmic reticulum stress-related TXNIP/NLRP3 activation-induced inflammation [78].

Mangiferin, delivered orally or via injection to rats at doses of 50 and 100 mg/kg for 5 weeks provided protection against doxorubicin (DOX) (15 mg/kg). Mangiferin reduced serum levels of lactate dehydrogenase (LDH) and creatine kinase, reduced MDA levels in plasma and cardiac tissue, and increased cardiac tissue levels of the antioxidant enzyme superoxide dismutase (SOD). Rats treated with doxorubicin (DOX) have significantly reduced necrotic foci, fibrotic area, and inflammatory cell number [79]. Mangiferin mitigates diabetic cardiomyopathy (DCM) and prevents the accumulation of myocardial collagen [80].

Isoproterenol-induced myocardial infarction is associated with imbalance of heart tissue and serum marker enzymes such as creatine phosphokinase (CPK), aspartate transaminase, alanine transaminase (ALT) and LDH, as well as enhanced levels of lipid peroxidation and histopathological changes. Mangiferin (10 mg/100 g BW) treatment for 4 weeks ameliorated effect of pH-induced pathological changes, reduced the formation of lipid peroxides, and retained the activity of cardiac markers [81].

Mangiferin has a significant role in the reduction of cholesterol, triglycerides, and free fatty acid (FFA) levels in both serum and heart, and can also enhance heart tissue phospholipid levels in isoproterenol-induced cardiotoxic rats [82]. Mangiferin preserved lysosomal integrity by decreasing the inflammatory process. Isoproterenol (two subcutaneous administrations of 200 mg/kg BW) treatment for 2 days significantly increased plasma TNF-α production as well as heart and serum lysosomal hydrolase activity, and also lowered the membrane stability. Mangiferin treatment (100 mg/kg BW) for 4 weeks prevented these alterations and restored the enzyme activity in rats [81].

## Oxidative stress

During the metabolic process, free radicals are produced in the human body, causing oxidation in biological macromolecules such as proteins, fatty acids, and nucleic acids. Free radicals at high concentration levels in the human body can cause oxidative stress, thus destroying internal redox balance and causing a variety of chronic diseases [83, 84].

Mangiferin oral administration (40 mg/kg/day) in diabetic rats for 30 days significantly reduced blood sugar, elevated plasma insulin levels, and increased antioxidant enzymes such as SOD, CAT and glutathione peroxidase (GPx) in the livers of diabetic rats compared to control rats. An additional reduction in glutathione (GSH) levels was also observed in the kidney [85]. In human glomerular renal endothelial cells, cadmium chloride promoted the secretion of two pivotal pro-inflammatory cytokines, IL-6 and IL-8, resulting in renal inflammation. However, mangiferin (75 μM) prevented cadmium-induced secretion of both both IL 8 and IL-6 by human glomerular endothelial cells, and can be used to prevent kidney inflammation [86].

Mangiferin protects against oxidative stress by regulating the production of NLRP3 & Nrf2, attenuating renal dysfunction and ameliorating morphological changes in CLP-induced septic mice, as well as lowering serum levels of IL-1β and IL-18, preventing tubular epithelial cells apoptosis, and suppressing the renal NLRP3 inflammasome activation in the kidneys [87]. Pal et al. 90] determined that orally administrated mangiferin (100 mg/kg BW) for 6 days slowered ROS formation, reduced ALT and alkaline phosphatase (ALP) concentrations, restored Pb^2+^-induced changes mitochondrial membrane potential, and regulated Bcl-2/Bax expression. Mechanistically, it also inhibited Pb(II)-induced activation of nuclear translocation of NF-κB, mitogen-activated protein kinases (MPAKs) (phosphor-ERK 1/2, phosphor-JNK phosphor-p38), and apoptotic cell death. Similarly, mangiferin considerably lowered the levels of total bilirubin, ALP, serum-glutamate pyruvate-transminase (SGPT), and serum-glutamate oxaloacetate-transminase (SGOT) [88].

Mangiferin has demonstrated a cytoprotective role against schizophrenia in a ketamine-induced rat model (50 miligram/kilogram, i.p., for seven days, twice a day). Mangiferin (10 to 100 μM for 7 days) treatment significantly prevented 6-hydroxydopamine (6-OHDA)-induced cell death in a concentration-dependent manner. It also significantly decreased the levels of IL-6 and MDA in brain tissues [89]. Wang et al. [90] found that concentrations of mangiferin (100, 200, and 400 mg/kg) enhanced the levels of SOD and NO in the BALF and serum of rats with chronic bronchitis, as well as decreased MDA levels, and content of TNF-α and IL-8 in lung tissues whereas the RAW264.7 macrophages COX-2 mRNA expression induced by LPS were also lowered.

In hyperuricemic mice induced by potassium oxonate, different concentrations of mangiferin (50, 100, and 200 mg/kg) lowered serum uric acid, urea nitrogen levels and creatinine concentration. Additionally, it also down regulated murine glucose transporter 9 (mGLUT9) and urate transporter 1 (mURAT1) mRNA and protein levels, and upegulated the murine organic anion transporter 1 (mOAT1). It lowered uric acid reabsorption and significantly increased renal organic cation levels, as well as carnitine transporter (mOCTN1, mOCTN2, mOCT1 and mOCT2) expression levels. Finally, it increased urinary and decreased kidney and serum mUMOD levels [91].

Mangiferin has protective role against the genotoxicity induced by cadmium chloride in Swiss albino mice. Treatment with a single intraperitoneal dose of mangiferin dose (2.5 mg/kg) significantly reduced the ratio of MnPCE and MnNCE, and increased the PCE/NCE ratio. Moreover, it also reduced lipid peroxidation and increased SOD, GSH, CAT, and GST activities in the liver [92]. Mangiferin has an effective role in N2A cells against 1-Methyl-4-phenyl-pyridine ion (MPP^+^-induced cyto-toxicity by restoring GSH content, and decreased the mRNA expression of both SOD and CAT [93].

Mangiferin (50 μM) exerted cytoprotection against magnesium chloride-induced toxicity by significantly increasing levels of SOD, GSH, CAT, GST, and by quenching free radicals, decreasing the percentage of HgCl(2) induced apoptotic cells, and normalized mitochondrial membrane potential and cellular antioxidant levels [94]. The application of Ca^2+^ increased mitochondria-generated ROS levels in the liver, but mangiferin treatment (40 miligram/kilogram BW) were treated in-vitro to Ca^++^ that prevented Ca^2+^-induced depletion of antioxidant enzymes and inhibited the production of free radicals in the liver [95].

## Neuroprotective role

Mangiferin has a cytotoprotective role against the neurotoxicity and cognitive impairment induced by aluminium chloride in male Swiss albino mice. Mangiferin (20 and 40 mg/kg) significantly reduced oxidative stress marker levels, as well as inflammatory cytokine levels and hippocampal brain-derived neurotrophic-factor (BDNF) content [96].

1-methyl-4-phenyl-1,2,3,6-tetrahydropyridine (MPTP) (30 mg/kg, i.p.) treatment enhanced oxidative stress, upregulated expression of Bax expression, apoptosis, protein expression and anti-apoptotic Bcl-2 marker in male C57BL/6 mice. Administration of mangiferin for 14 days (10, 20 and 40 mg/kg) prevented dopamine depletion, and MPTP-induced behavioral deficits [97].

It has been shown that mangiferin (0.1–10 μM) inhibits the contraction-induced tracheal stimuli in a concentration-dependent manner, such as 5-hydroxytryptamine, carbachol, histamine or allergen. Pre-contracting of carbachol by mangiferin also caused a significant relaxation of the tracheal ring, indicating that the property of having anti-contractile and relaxing properties was avoided by removal of the epithelium. Mangiferin effect was inhibited by the inhibitor of 1H-[1, 2, 4] oxadiazolo [4,3-a]quinoxaline-1-one (ODQ) (10 μM), inhibitor of nitric-oxide synthase, Nω-nitro-L-arginine methyl ester (L-NAME) (100 μM), inhibitor of soluble guanylate cyclase and inhibit the [4,3- α]quinpxalin-1-one (ODQ) (10 μM), but not inhibitors of adenylate cyclase such as 9-(tetra-hydro-2-furyl) adenine (SQ22536) (100 μM). Antispasmodic effects of mangiferin were also sensitive to potassium-channel blockers, for example tetraethylammonium (TEA), apamin and glibenclamide. In addition, Ca++-induced contractions in K+ (60 mM)-depolarized tracheal-ring arrangements were inhibited by mangiferin. Furthermore, in cultured tracheal rings, mangiferin increased protein levels of cGMP and nitric oxide synthase at the cellular level. Finally, the mangiferin abolished the increase in cGMP levels induced by co-incubation with -1H- [1, 2, 4] oxadiazole, [4,3-a] quinoxaline-1-one, or L-NAME [98].

The mangiferin oral administration (10 and 20 miligram/kilogram daily) enhanced cellular responses, antigen-specific IgM levels, and lymphoid organ weights in cyclophosphamide-treated male Wistar rats after 2 weeks. It also reduced lipid peroxidation, lymphocytes amount, macrophages, and polymorphonuclear cells, as well as SOD and CAT activity [2].

Mangiferin provided protection against neuroinflammation and oxidative damage in the brains of young male Wistar rats. Different concentrations of mangiferin (15, 30, and 60 mg/kg) prevented from (1) enhanced IL-1β and glucocorticoid (GC) plasma levels, (2) loss of redox balance and reduction in catalase brain levels, (3) increase in pro-inflammatory mediators such as TNF-α, NF-κB, TNF receptor 1, as well as synthesis enzymes such as iNOS and COX-2, and (4) increased in lipid peroxidation. These aspects of the protective effect proved mangiferin use may be a novel strategy for treatment of neuropsychiatric/neurological pathologies [99]. In another study conducted by [100], oral administration of mangiferin (10, 50, or 100 mg/kg) increased novel object recognition when immediate post-training was given. Cell proliferation stimulated by mangiferin and it is induced a significant-increase in the level of supernatant of nerve growth factor and TNF-alpha, as well as greater levels of cytokines and neurotrophic factors, in human U138-MG glioblastoma cells.

## Hyperlipidemia

Mangiferin is protective against hyperlipidemia through the regulation of metabolic pathways such as the glyoxylate, tricarboxylic acid (TCA)and taurine cycles, as well as metabolism of dicarboxylate, glycerophospholipid, hypotaurine, and threonine, and serine, glycine, and primary bile acid biosynthesis [101]. Likewise, mangiferin enhances proteins which lead to mitochondrial biogenesis and oxidative activity such as cytochrome c oxidase subunit 6B1 (Cox6b1) and oxoglutarate dehydrogenase E1 (DHTKD1). It also lowers the levels of proteins which are critical for lipogenesis, such as acetyl-CoA carboxylase 1 (Acac1) gene and fatty acid stearoyl-CoA desaturase 1 (Scd1), and upregulates proteins important to bioenergetics, downregulates mitochondrial proteins which control *de novo* lipogenesis, and prevents adiposity [102]. In human neuroblastoma SK-N-SH cells, mangiferin considerably increased cell viability, improved mitochondrial membrane potential, and reduce apoptosis induced by rotenone [103].

Mangiferin also enhanced muscle glucose oxidation in high fat diet (HFD)-fed mice without altering fatty acid oxidation. In cultured C2C12 myocytes, mangiferin increased glucose and pyruvate oxidation and ATP production, without affecting the oxidation of fatty acids. Furthermore, it suppressed the conversion of pyruvate to lactate via anaerobic metabolism, but increased the oxidation of pyruvate [104].

Total FFA levels, final body weight, serum TG levels, visceral fat-pad weight, liver weight, muscle and hepatic total FFA content, and hepatic TG levels were all significantly decreased by administration of mangiferin (50 and 150 mg/kg) in hamsters. Mangiferin upregulated mRNA expression of PPAR-α, carnitine palmitoyltransferase 1 (CPT-1), and fatty acids (CD36), but decreased the mRNA expression of sterol regulatory element binding protein 1C (SREBP-1c), diacylglycerol acyltransferase 2 (DGAT-2), microsomal triglyceride transfer protein (MTP) and acetyl coenzyme a carboxylase (ACC) in the liver. Mangiferin was also stimulated the expression of mRNA of CPT-1, PPAR-α, lipoprotein lipase (LPL) and CD36 in muscle [15].

It also decreased the production of reactive oxygen species through both mitochondria isolated from the LDLr (-/-) liver and lymphocytes from the spleen. It also protects the depletion of mitochondrial substrates NAD(P)H-bound and spontaneous-oxidation NAD(P)H [105]. In another study, mangiferin (50, 100, 150 mg/kg) treatment for 6 weeks lowered FFA and TG levels in the plasma and liver of hyperlipidemic rats and increased levels of β-hydroxybutyrate. It markedly increased FFA uptake and markedly reduced intracellular accumulation of FFA and TG in HepG2 cells. Mangiferin knowingly increased phosphorylation of AMP – AMPK (activated protein kinase) and its proteins downstream comprises carnitine palmitoyltransferase 1 (CPT1) and fatty acid translocase (CD36), but significantly reduced the expression of acyl - CoA: diacylglycerol acyl transferase Enzyme 2 (DGAT2) and activity of acetyl-CoA carboxylase (ACC), by increasing the phosphorylation levels in their in vivo and in vitro studies [106].

Mangiferin up-regulates BMP-4, BMP-2 (bone-morphogenetic protein) and TGF-β (transforming growth factor) expression and several key chondrogenesis markers, including the identification of region Y-box, (SRY box) mesenchymal stem cells containing the gene 9 (SOX9), ALPHA1 collagen type 2 (col2 alpha1), aggrecan and connexin cartilage. BMP-2, BMP-4, SOX9, Col2alpha1, binding proteins and aggrecan and matrix metalloproteinase (MMP) -MMP-13 were produced by mangiferin in the MSC stimulated with IL-1β and disintegrin and metalloproteinases with thrombospondin motifs (ADAMTS5). Mangiferin up-regulated the Smad 2, Smad 3, Smad 1/5/8, and SOX9 phosphorylation in IL-1β-stimulated MSCs. SOX9 siRNA inhibited the Smad 2, Smad 3, Smad 1/5/8, Col2α1 and aggrecan expression activation by the presence of mangiferin, In summary, chondroprotective and chondrogenic effects are executed by mangiferin on scratched MSCs and it also mediates these effects via pointing the SOX9 and Smad pathways in multiple aspects. [107, 108].

## Miscellaneous properties

Mangiferin exerted protective effects against atopic dermatitis, bronchial asthma, and other allergic diseases. It also inhibited IgE production, anaphylaxis reaction, histamine-induced vascular permeability, histamine release, and the lymphocyte proliferative response in rats. Furthermore, it lowered the amount of B and T lymphocytes, which are responsible for allergic responses [109]. Mangiferin administration significantly reduced alveolar bone loss (ABL) through inhibition of COX-2 expression and leukocyte rolling and adhesion, while maintaining normal levels of lipoxin A4 [110].

Mangiferin provided a shielding effect against the damaging effect of hydrogen peroxide in human keratinocytes (HaCaT). It also neutralized free radicals, including oxyl, peroxyl, and superoxide radicals. These radicals regulate the expression and activation of the plasminogen protease gene, and mangiferin inhibited MMP-1 gene and protein expression levels. It also inhibited DNA binding activity of activator protein-1 (AP-1), a transcription factor for MMP-1, down stream of ERK and JNK [111]. Treatment with mangiferin (50 mg/kg) resulted in significant reduction of airway inflammation in peripheral blood vessels and bronchial inhibition of IL-4 and IL-5 in bronchoalveolar lavage fluid for 24 days and lymphocyte culture supernatant [109]. Mangiferin also protected normal human intestinal epithelial cells (HIECs) from radiation-induced injuries by modulating their genotoxic effects and eliminating 46.8% of the total double strand breaks (DSBs) of the cells which were exposed to 2 Gy ionizing irradiation (IR) [112]. Significant reduction had been seen in TNF-α (tumor necrosis factor) levels tempted by inhibited nuclear factor-kappa-B stimulation in scopolamine in mice brain or TNF stimulated cells by using mangiferin (20 mg/kg). Additionally, mangiferin enhances the deficits of long-term cholinergic-memory by inhibiting stimulation of cholinergic receptor and NF-κB activation suppression [113].

Mangiferin inhibits bone resorption and osteoclast formation by weakening RANKL-induced signaling. It also decreased expression of osteoclast gene markers such as calcitonin receptor, cathepsin K, V-ATPase d2 and DC-STAMP. Mechanistically, mangiferin suppressed RANKL-induced activation of NF-κB, as well as p65 nuclear translocation and IκB-α degradation. Moreover, inhibitory effect of mangiferin on induction of ERK phosphorylated RANKL [114]. The administration of galactosamine (GAL) (400 mg/kg) in rats showed the elevation in ALP, ALT, triglycerides level, lipid-peroxidation, total cholesterol and reduction of total proteins, serum albumin and cellular GSH. Meanwhile, in GAL treated rats, mangiferin, owing to its antioxidant defense mechanisms, significantly altered GAL-induced adverse effects, and suppressed the Nrf2 pathway, reduced inflammation, and inhibited NFκB activity [115].

Mangiferin possesses antibacterial activity against gram-positive (i.e. *Bacillus pumilus*) and gram-negative bacteria (i.e. *Salmonella agona*). It also protects from the harmful effects of. *Enterococci* and *Mycobacterium tuberculosis*, and showed antifungal effects against *trichoderma reesei,, aspergillus flavus,* and *Thermoascus aurantiacus* [116, 117]. It also acts as a curative and preventive agent against periodontal disease, as well as free radical-mediated oxidative damage in neurons, cardiac muscle, liver, and kidney tissue [110, 118]. It also exhibited good anti-HIV-1 activity through inhibition of the HIV-1 protease, and lowered intestinal neoplasms in rats and was active against herpes simplex virus (HSV-1) [8, 119]. Mangiferin oral supplementation (50 mg/kg) suppressed the growth of nematode *Trichinella spiralis*. It inhibited themast cell degranulation, lowered the serum levels of specific anti-*Trichinella* IgE and declined the number of parasitic larvae through out the life cycle of parasite [3, 120].

Mice were challenged with LPS (0.83 mg/kg, i.p.), which induced oxidative stress. Different concentrations of mangiferin (20 and 40 mg/kg) significantly ameliorated anxiety-like behaviour and also improved anhedonic behaviour. It also enhanced glutathione concentrations, CAT and SOD activity, and reduced lipid peroxidation and nitrite levels in the hippocampus and the prefrontal cortex. It also attenuated neuroinflammation in the prefrontal cortex and hippocampus by reducing IL-1β levels [121].

## Conclusions

Mangiferin is a bioactive compound that demonstrates many health perspectives and has been used to prepare medicinal and food supplements. Owing to the presence of mangiferin in leaves, bark, seed, peel, flowers, and pulp, a potential source of dietary polyphenols with elevated antioxidative properties could be available for industrial purposes. The phenolic compounds present in the mango peel are greatly affected by the geographic locations of the plants. Mangiferin has also been demonstrated to exert protective effects against degenerative diseases such as atherosclerosis, cancers (i.e. breast, colon, neural, skin and cervical), obesity and diabetes. It also protects the body against damage associated with oxidative stress. There should be more clinical trials by using this miracle bioactive compound in future.
